# Neurons Are a Primary Driver of Inflammation via Release of HMGB1

**DOI:** 10.3390/cells10102791

**Published:** 2021-10-18

**Authors:** Huan Yang, Ulf Andersson, Michael Brines

**Affiliations:** 1 Institute for Bioelectronic Medicine, The Feinstein Institutes for Medical Research, 350 Community Drive, Manhasset, NY 11030, USA; brinesmichael@gmail.com; 2 Department of Women’s and Children’s Health, Karolinska Institute, Karolinska University Hospital, 17176 Stockholm, Sweden

**Keywords:** high mobility group box 1, TRPV1, nociception, cytokine, inflammation, nerve injury, arthritis

## Abstract

Recent data show that activation of nociceptive (sensory) nerves turns on localized inflammation within the innervated area in a retrograde manner (antidromically), even in the absence of tissue injury or molecular markers of foreign invaders. This neuroinflammatory process is activated and sustained by the release of neuronal products, such as neuropeptides, with the subsequent amplification via recruitment of immunocompetent cells, including macrophages and lymphocytes. High mobility group box 1 protein (HMGB1) is a highly conserved, well characterized damage-associated molecular pattern molecule expressed by many cells, including nociceptors and is a marker of inflammatory diseases. In this review, we summarize recent evidence showing that neuronal HMGB1 is required for the development of neuroinflammation, as knock out limited to neurons or its neutralization via antibodies ameliorate injury in models of nerve injury and of arthritis. Further, the results of study show that HMGB1 is actively released during neuronal depolarization and thus plays a previously unrecognized key etiologic role in the initiation and amplification of neuroinflammation. Direct targeting of HMGB1 is a promising approach for novel anti-inflammatory therapy.

## 1. Introduction: Activation of Nociceptive Neurons Turns on Inflammation

**Figure 1:**
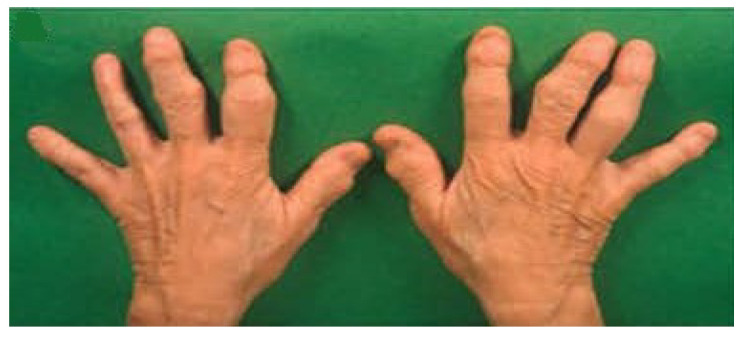
Digital nerve loss protects against arthritis-induced joint destruction. Swelling and joint deformity exist for distal and proximal interphalangeal joints except for the fourth digit in a patient suffering from long-standing psoriatic arthritis. She had previously suffered a complete traumatic transection of the fourth digital nerve as a child, before the onset of arthritis, which had resulted in sensory denervation (Reproduced from Kane et al. [4]).

Recent studies have shown that activation of nociceptors (TRPV1+) in the absence of tissue damage or pathogen-related products fully mobilizes local innate immune responses. For example, using optogenetic techniques, Cohen et al. have reported that sterile triggering of cutaneous nociceptors produces a sustained multicellular immune response within the innervated tissue via the antidromic nerve reflex arc [1]. To underscore the critical role of neurons in the process, this biological response has been termed neuroinflammation. Many different processes which stimulate sensory neurons can also simultaneously activate inflammatory processes and result in acute and chronic diseases. As one example, immune-antibody complexes of cartilage collagen within the joint space activate sensory neurons via Fc receptors for IgG resulting in pain behavior before the development of inflammation and damage within the joint [2]. Interfering with neuronal activation greatly attenuates subsequent joint inflammation, underscoring the importance of neuroinflammation in the pathophysiology [3]. Notably, clinical experience also supports a critical role for sensory nerves in the development of inflammatory diseases. As one example, Kane et al. have reported that a patient with psoriatic arthritis who had suffered nerve transection of the fourth finger prior to contracting the disease, was completely protected from the development of inflammation and joint deformity in that digit in contrast to the other fingers (Figure 1) [4]. Furthermore, an experimental arthritis study designed to investigate a possible relationship between joint innervation and joint inflammation demonstrated that prior sensory denervation, with preserved motor function, prevented the development of arthritis in the denervated knee [4]. Importantly, persistent activation or malfunction of the nociceptive system also gives rise to chronic pain [5]. As reviewed by Peirs and Seal, noxious stimuli activate primary sensory neurons that transmit nociceptive information to the spinal and medullary dorsal horns. A cascade of signaling subsequently leads to neuroinflammation and perceived pain [6].

The current paradigm of the pathophysiology of neuroinflammation has focused on the observation that stimulated sensory neurons actively release a variety of vasoactive and immune stimulating molecules, typically histamine and neuropeptides [7]. These mediators act synergistically with local immunocompetent cells, e.g., Langerhans cells, to establish a mature inflammatory complex ultimately culminating in the accumulation of neutrophils and lymphocytes, generally associated with secondary tissue damage and maintenance of a state of disease. However, neurons also actively restrict the local inflammatory response via monitoring by the vagus nerve and the central nervous system. Based on this information, the central nervous system exerts homeostatic controls via the autonomic nervous system and the hypothalamic-pituitary-adrenal system [8].

## 2. Role of High Mobility Group Box 1 Protein (HMGB1) in the Inflammatory Response

In addition to the molecular products well-known for release from sensory neurons, other important molecules have been implicated in the immune response. One key player in activating inflammation is HMGB1, which is an evolutionarily highly conserved, nuclear, non-histone DNA binding protein present in all nucleated cells [9]. Prior work has shown that HMGB1 is passively released during lytic cell death processes, as well as secreted by activated innate immune cells. Certain post-translationally modified isoforms of HMGB1 operate extracellularly as proinflammatory mediators in infectious and sterile inflammatory conditions [10,11,12]. Further, HMGB1 levels in the central nervous system and in dorsal root ganglion (DRG) cells are elevated in many neuronal injury models including tibial nerve injury [13], stress-induced headache [14], ischemic brain damage [15], cocaine exposure [16], ethanol overdose [17], morphine-mediated analgesic tolerance and hyperalgesia [18], and subarachnoid hemorrhage [19]. Stressed and damaged neurons collaborate with activated microglial cells and astrocytes to further propagate inflammation via proinflammatory molecules including HMGB1. However, important questions regarding the functional role of neuronal HMGB1 in the cellular collaboration driving inflammation in the central nervous system or in the periphery have, until lately, remained elusive. One important, incompletely investigated area is assessing the importance of neuronal HMGB1 in inflammation and whether its release occurs only via neuronal injury, or whether, similar to immune cells, uninjured neurons can directly release HMGB1 to initiate immune responses. Here, we focus on reviewing recent findings addressing these issues.

## 3. HMGB1 Is Actively Released by Nociceptive Neurons

Nuclear HMGB1 is ubiquitously expressed in the central nervous system including neurons, satellite cells, Schwann cells, microglia, and astrocytes, and the intracellular levels are further enhanced during traumatic neuropathy, denoting a possible link between HMGB1 release and nociception [20]. A programmed nuclear-cytoplasmic translocation of HMGB1 is a prerequisite in innate immune cells for active HMGB1 release via exocytosis of cytoplasmic vesicles such as secretory lysosomes [21] or via exosomal release during sepsis [22]. Studies by Merianda et al. provided direct evidence that, in mice with sciatic nerve injury, HMGB1 located within DRGs shifts from cell bodies towards release [23]. These investigators used L4–L5 DRG neurons cultured from animals that had been conditioned 7 d previously by unilateral sciatic nerve crush. HMGB1 protein shifts from being cell body-predominant in the naive neurons to being axon-predominant in the injury-conditioned neurons. Immunofluorescent images of cultured neurons showed higher cell body signals in naive neurons and higher axonal signals in injury-conditioned neurons. Hence, this study provided direct evidence that during injury, neuronal HMGB1 shifts from cell bodies to axons towards release. All these findings implicate that HMGB1 is actively released from neurons to elicit extracellular events.

**Figure 2:**
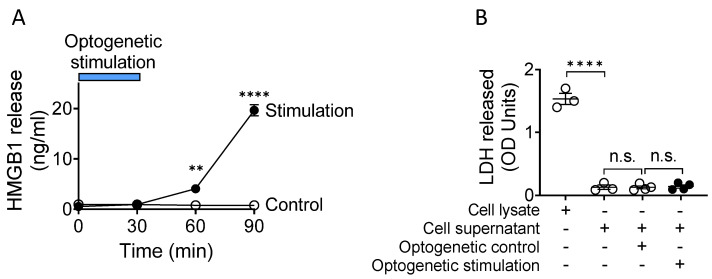
Stimulated neurons actively release HMGB1. (**A**). Sensory neurons harvested from dorsal root ganglia (DRG) of Vglut2-Cre/ChR2-eYFP mice were cultured and exposed to (470 nm; stimulates) or yellow light (595 nm, not-stimulating control) at 20 Hz, and 10% duty cycle for 30 min using optical fiber-coupled LEDs. Blue bar indicates the duration of stimulation. A time-dependent increase in secreted HMGB1 levels is observed following optogenetic stimulation (at 60 min: **: *p* < 0.01, at 90 min: ****: *p* < 0.0001); reproduced from Yang et al. [27]. (**B**). DRG viability was assessed by release of the soluble cytoplasmic enzyme lactate dehydrogenase (LDH), which leaks out following membrane disruption. Cell lysate was included as positive control. No significant increase in cell death is observed following optogenetic stimulation. *n* = 3–4 separate experiments, and each performed in duplicate (****: *p* < 0.0001), n.s.: not significant; reproduced from Yang et al. [27].

Although neurons do not contain secretory lysosomes, studies have implicated that HMGB1 is actively released from excessively (hyper) depolarized neurons in response to optogenetic stimulation [24], from neurons stimulated by TNF [25] or following ethanol exposure [26]. Using an optogenetic approach, we directly demonstrated that stimulated sensory neurons actively release HMGB1 [27]. Specifically, to selectively activate sensory neurons, we generated transgenic Vglut2-Cre/ChR2-eYFP mice which express channelrhodopsin-2 (ChR2) coupled to an enhanced yellow fluorescent protein (ChR2-eYFP) directed by vesicular glutamate transporter type 2 (VGlut2) promoter. ChR2 is a light-gated ion channel that is activated by exposure to blue light. Previous work has shown that activation of these light-gated channels depolarizes the DRG neurons and elicits propagated action potentials [28]. Vesicular glutamate transporter 2 (VGlut2) is expressed by peripheral glutamatergic sensory neurons. Sensory neurons, harvested from dorsal root ganglia of Vglut2-Cre/ChR2-eYFP mice, were then cultured and stimulated by 470 nm blue light in vitro and subsequently expressed significant levels of cytoplasmic HMGB1 in contrast to unstimulated DRGs. We subsequently observed (Figure 2A) a time-dependent increase in the extracellular HMGB1 concentrations after the optogenetic stimulation [27]. In contrast, photo-stimulation of DRG sensory neurons using yellow light (595 nm), which does not activate ChR2, failed to induce HMGB1 release. These combined results thus indicate that stimulated nociceptors actively translocate nuclear HMGB1 to the cytoplasm for ultimate release at the nerve ending. Lactate dehydrogenase (LDH), a soluble cytoplasmic enzyme released upon cell membrane disruption, was not released during light exposure, further confirming that HMGB1 is actively secreted by stimulated nociceptors, and not passively released via lytic cell death [27] (Figure 2B). In summary, although the full temporal profile of HMGB1 release in response to neuronal activation remains to be determined in future experiments, the results of these studies definitively show that, as with immune cells, sensory neurons can actively release HMGB1 in response to stimulation. That this release is physiologically relevant is shown using neuronal HMGB1 knock-out and neutralization in preclinical models, as discussed below.

## 4. HMGB1 Induces Nociceptive Responses via Neuronal TLR4-Dependent Mechanisms

The biological activities of HMGB1 depend upon receptor binding which is sensitive to the redox state of each of HMGB1′s three cysteines [11]. Fully reduced (all-thiol) HMGB1 exerts chemotactic activity by forming a heterocomplex with the chemokines CXCL12 and CXCR4, which initiates recruitment of inflammatory cells in a synergistic fashion, compared to CXCL12 alone [29,30]. In contrast, the cytokine-stimulating activity of HMGB1 requires cysteine 23 and 45 to form a disulfide link as a result by mild oxidation, while keeping the C106 residue in its reduced state [31]. This disulfide isoform exclusively binds and activates the TLR4/MD-2 complex [32,33]. Finally, HMGB1 with any of the cysteines terminally oxidized (sulfonyl HMGB1) has until recently been regarded as an immunologically inactive molecule. However, recent studies indicate that sulfonyl HMGB1 is actually a potent anti-inflammatory molecule [34].

**Figure 3:**
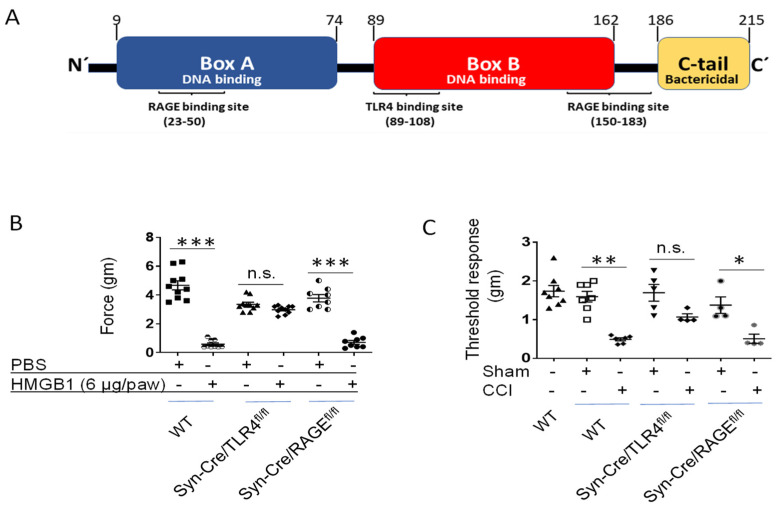
Neuronal TLR4, not RAGE, mediates HMGB1-induced nociceptive response. (**A**). Location of receptor binding sites in the HMGB1 molecule. The human HMGB1 protein expresses 214 amino acid residues and contains three functional domains: two DNA binding regions termed Box A and Box B, and a bactericidal C-terminal tail. The three ligand binding sites of the HMGB1 protein: RAGE binding site (23–50 aa’), TLR4 binding site (89–108 aa’) [31] and RAGE binding site (150–183 aa’). (**B**). Wild type (C57BL/6), Syn-Cre/TLR4^fl/fl^ and Syn-Cre/RAGE^fl/fl^ mice (generated by crossing Rage ^fl/fl^ (from Dr. Daolin Tang, Dallas, TX, USA) or TLR4 ^fl/fl^ (Jackson Laboratories, Hudson, NY, USA) to mice carried the Synapsin I promoter-driven Cre recombinanse transgene (Jackson Laboratories)) had intra-plantar injection of HMGB1 (6 µg/paw) or vehicle (PBS) on hindpaw, and 5 h later mechanical hypersensitivity (dynamic plantar aesthesiometer) was assessed. (*n* = 8–10 mice per group. ***: *p* < 0.0001). n.s.: not significant. (**C**). Wild type (WT), Syn-Cre/TLR4^fl/fl^ and Syn-Cre/RAGE^fl/fl^ mice (male, 8–12 weeks old) were subjected to sciatic nerve ligation surgery (CCI) or sham surgery. Two weeks after sciatic nerve ligation (or sham) surgery, mechanical hypersensitivity (von Frey filament) was assessed. *n* = 4–8 per group. *: *p* < 0.02. **: *p* < 0.001. n.s.: not significant.

The HMGB1 molecule has binding sites for both the receptor for advanced glycation end-product (RAGE) and Toll-like receptor 4 (TLR4; Figure 3A). Using global TLR4 knockout (KO) mice, Svensson et al. demonstrated that TLR4 is required for HMGB1-mediated hyperalgesia [35,36]. However, no previous study has investigated the impact of tissue-specific TLR4 in neuronal HMGB1 signaling. To gain insight into the mechanism of neuronal HMGB1-induced nociceptive responses, we generated novel neuronal TLR4 (Syn-Cre/TLR4^fl/fl^) and RAGE-specific KO (Syn-Cre/RAGE^fl/fl^) mouse models. To determine if the absence of neuronal TLR4 is sufficient to significantly reduce hyperalgesia in the presence of high levels of HMGB1, mice had paw injections of disulfide HMGB1 (6 µg/paw) and mechanical hypersensitivity was assessed 5 h afterwards. Similar to global KO, neuronal specific TLR4 KO had significant protection against HMGB1-induced allodynia as compared to wild type or neuronal RAGE KO mice (Figure 3B). In agreement with this observation, mice subjected to sciatic nerve injury developed hypersensitivity in wild type and neuronal RAGE KO as compared to sham-operated controls. In contrast, neuronal TLR4 KO mice were significantly protected from this sciatic injury-induced allodynia (Figure 3C). Taken together, these findings show that HMGB1-inducing nociceptive responses predominantly occur via a neuronal TLR4-dependent signaling mechanism. The effect of neuronal knock-out of HMGB1 on TLR4 expression, if any, is currently unknown.

## 5. Neuronal HMGB1 Ablation/Neutralization Reduces Inflammation and Hyperalgesia

**Figure 4:**
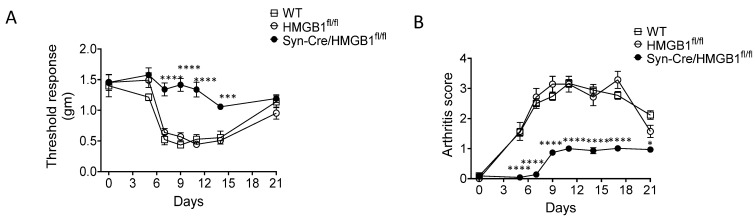
Ablation of neuronal HMGB1 reduces joint inflammation and cartilage destruction, while improving hyperalgesia in murine collagen antibody-induced arthritis. Polyarthritis was induced by administration of anti-collagen antibodies in mice. Wild type (WT) and HMGB1^fl/fl^ control mice develop increased mechanical hypersensitivity (**A**) and polyarthritis (**B**). A significantly delayed onset and reduced severity of polyarthritis are observed in HMGB1 knock out (Syn-Cre/HMGB1^fl/fl^) mice. This is accompanied by a marked reduction in mechanical hyperalgesia. (Syn-Cre/HMGB1^fl/fl^ versus HMGB1^fl/fl^ group: * *p* < 0.05, *** *p* < 0.001, **** *p* < 0.0001. Reproduced from Yang et al. [27]).

The functional in vivo role of nociceptors and HMGB1 in inflammation was recently evaluated in studies of mice with HMGB1 expression silenced only in neurons [27]. Using mice expressing Cre recombinase under the control of neuronal-specific synapsin promoter, and by crossing synapsin-Cre (Syn-Cre) mice with floxed HMGB1 mice (HMGB1^fl/fl^), we have generated mice with neuronal-specific ablation of HMGB1 (Syn-Cre/HMGB1^fl/fl^). This strain of mice allows us to determine the effects of selective neuronal HMGB1 deficiency in neuroinflammation. Standardized sciatic nerve injury which generates severe neurogenic inflammation in wild type mice had a much milder course of cutaneous paw inflammation and allodynia in the neuronal-specific HMGB1 knock out (Syn-Cre/HMGB1^fl/fl^) animals (Figure 4A). Further, experimental collagen antibody-induced arthritis (CAIA) in wild-type mice induces painful, destructive polyarthritis that depends on nociceptor-induced neuroinflammation [37,38]. The onset of CAIA in HMGB1 lacking Syn-Cre/HMGB1^fl/fl^ mice was significantly delayed compared to that in controls and the neuronal HMGB1 knockout mice were subsequently substantially protected from joint inflammation and allodynia (Figure 4B). Thus, nociceptor HMGB1 is an essential mediator of the neuroinflammatory response to different forms of tissue injury.

HMGB1-specific monoclonal antibodies have been demonstrated to be effective for the treatment of a wide range of neuroinflammatory diseases in multiple preclinical models, including stroke [39,40,41,42], traumatic brain injury [43], Parkinson’s disease [44], epilepsy [45,46], autoimmune encephalomyelitis [47,48], Alzheimer’s disease [24], nerve root compression [49], and cognitive decline after sepsis or major surgery [50,51]. Potential therapeutic implications have been addressed in the comprehensive recent reviews of Paudel et al. [52] and Nishibori et al. [53,54].

Interestingly, the therapeutic results seem to be more consistent and successful in disease models where neuronal HMGB1 is involved than in non-neuronal HMGB1-dependent inflammatory conditions. The reasons for these discrepant therapeutic outcomes are presently not fully understood, but may depend upon the molecular form of HMGB1. Specifically, extracellular HMGB1 either generates inflammation by operating as an individual molecule that signals via TLR4 or the receptor for advanced glycated end products (RAGE) or, alternatively, by acting complex-bound to extracellular DAMP/PAMP molecules. These complexes are endocytosed via RAGE expressed on macrophages and other innate immunity cells and end up in the endolysosomal system of these cells. There, during acidic conditions, HMGB1 acts as a detergent and damages the lysosomal membrane allowing partner molecules access to cytosolic sensors including inflammasomes that initiate inflammation [55]. Extracellular HMGB1 has a vigorous capacity to form these complexes with partner molecules during systemic inflammatory conditions. From a therapeutic point of view, this biology may create major obstacles for HMGB1-specific antagonists to recognize and neutralize HMGB1. However, the neuronally released HMGB1 can be discharged close to its cognate TLR4 receptors with much less risk that HMGB1 will first bind to other molecules, causing steric hindrance for HMGB1-specific antagonists to mediate beneficial therapeutic effects. We thus speculate that future treatment with HMGB1-specific antagonists might offer a unique clinical opportunity to pacify HMGB1 in diseases where harmful neuronal HMGB1 release is at hand. Many scientists and clinicians interested in HMGB1 are puzzled and disappointed by the fact that, after more than two decades of HMGB1 research, we still lack successful therapeutic HMGB1 antagonists in clinical use. If our speculations about problems and opportunities for HMGB1-specific antibody treatment are correct, they might be helpful to optimize future clinical therapeutic strategies.

## 6. Perspective

**Figure 5:**
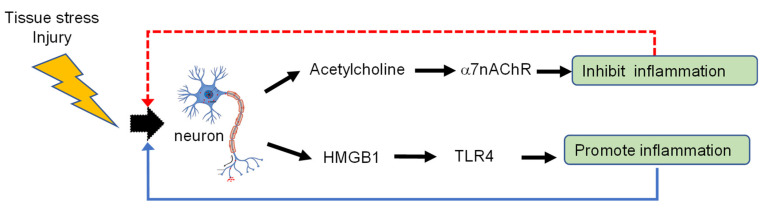
Neurons regulate the inflammatory milieu. Neurons both activate inflammation via release of HMGB1 and other pro-inflammatory molecules, as well as inhibit inflammation via acetylcholine release from vagal nerve fibers (the inflammatory reflex) [60]. However, these actions also feedback upon neurons to provide inhibition (red dashed line) to counter-balance further amplification (blue solid line) of inflammation. The discovery that neuronal HMGB1, a well characterized endogenous pro-inflammatory mediator, is a fundamental driver of the cascade of immune system defense provides a mechanism by which the nervous system provides yin–yang control of anti- and pro-inflammatory factors to maintain homeostasis.

Studies of nociception and neuropathic pain have revealed that HMGB1 release from injured or activated cells occurs as it translocates from the nucleus to the cytosol as a mechanism of active release [35,56,57,58]. However, it was not previously known whether HMGB1 is actively released by neurons during neuropathic inflammatory pain syndromes. Our critical recent findings not only confirmed the role of actively released HMGB1, but also established that neuronal HMGB1 is required to induce neuroinflammation in tissues [27]. The active involvement of neurons in the initiation of inflammation and tissue damage is direct evidence that the nervous system can stimulate innate immune responses and inflammation. It has been long established that neurotransmission in the vagus nerve inhibits inflammation and nociceptive responses through “the inflammatory reflex” [59,60]. Successful preclinical as well as clinical studies with implanted or external vagus nerve stimulators in patients with rheumatoid arthritis or inflammatory bowel disease have validated the importance of this mechanism [61,62,63,64,65,66]. The new observation that neurons directly release HMGB1 as the trigger of immune system defense during tissue injury, while simultaneously directing anti-inflammatory activities via the inflammatory reflex, establishes an important framework to understand how the nervous system evolved to reflexively enhance or inhibit inflammation. The ability to inhibit and stimulate inflammation yields a highly controllable homeostatic process to modulate inflammation to optimize survival and propagation of the species against threat from infection and injury (Figure 5). This new understanding of the neural reflex basis of immune homeostasis, and revelation of underlying mechanisms for the neural control of inflammatory signaling, paves the way for the development of experimental therapeutics, e.g., specific HMGB1 antagonists, or of electromagnetic devices to block neuronal HMGB1 release as novel treatments for inflammatory diseases.

## Abbreviations

CAIA: collagen antibody-induced arthritis; CCI: chronic constriction injury; CNS: central nervous system; HMGB1: high mobility group box 1; RAGE: receptor for advanced glycation end-product; TLR4: Toll-like receptor 4; and TRPV1: transient receptor potential vanilloid type 1.
